# Rehabilitation strategies following oesophagogastric and Hepatopancreaticobiliary cancer (ReStOre II): a protocol for a randomized controlled trial

**DOI:** 10.1186/s12885-020-06889-z

**Published:** 2020-05-13

**Authors:** Linda O’Neill, Emer Guinan, Suzanne Doyle, Deirdre Connolly, Jacintha O’Sullivan, Annemarie Bennett, Grainne Sheill, Ricardo Segurado, Peter Knapp, Ciaran Fairman, Charles Normand, Justin Geoghegan, Kevin Conlon, John V. Reynolds, Juliette Hussey

**Affiliations:** 1 Discipline of Physiotherapy, School of Medicine, Trinity College, the University of Dublin, Dublin, Ireland; 2 School of Medicine, Trinity College, the University of Dublin, Dublin, Ireland; 3 School of Biological and Health Sciences, Technological University Dublin, Dublin, Ireland; 4 Discipline of Occupational Therapy, School of Medicine, Trinity College, the University of Dublin, Dublin, Ireland; 5 Department of Surgery, Trinity Translational Medicine Institute, Trinity College, the University of Dublin and St. James’s Hospital, Dublin, Ireland; 6 Department of Clinical Medicine, Trinity College, the University of Dublin, Dublin, Ireland; 7 Centre for Support and Training in Analysis and Research, and School of Public Health, Physiotherapy and Sports Sciences, University College Dublin, Dublin, Ireland; 8 Department of Health Sciences and the Hull York Medical School, University of York, York, UK; 9 Exercise Medicine Research Institute, School of Medical and Health Sciences, Edith Cowan University, Joondalup, Australia; 10 Centre for Health Policy and Management, Trinity College, the University of Dublin, Dublin, Ireland; 11 Department of Surgery, St Vincent’s University Hospital, Dublin, Ireland; 12 Department of Surgery, Tallaght University Hospital, Dublin, Ireland; 13 Department of Surgery, Trinity College, the University of Dublin, Dublin, Ireland

**Keywords:** Oesophagogastric cancer, Pancreatic cancer, Liver cancer, Hepatobiliary cancer, Multidisciplinary rehabilitation, Exercise, Diet

## Abstract

**Background:**

Curative treatment for upper gastrointestinal (UGI) and hepatopancreaticobiliary (HPB) cancers, involves complex surgical resection often in combination with neoadjuvant/adjuvant chemo/chemoradiotherapy. With advancing survival rates, there is an emergent cohort of UGI and HPB cancer survivors with physical and nutritional deficits, resultant from both the cancer and its treatments. Therefore, rehabilitation to counteract these impairments is required to maximise health related quality of life (HRQOL) in survivorship. The initial feasibility of a multidisciplinary rehabilitation programme for UGI survivors was established in the Rehabilitation Strategies following Oesophago-gastric Cancer (ReStOre) feasibility study and pilot randomised controlled trial (RCT). ReStOre II will now further investigate the efficacy of that programme as it applies to a wider cohort of UGI and HPB cancer survivors, namely survivors of cancer of the oesophagus, stomach, pancreas, and liver.

**Methods:**

The ReStOre II RCT will compare a 12-week multidisciplinary rehabilitation programme of supervised and self-managed exercise, dietary counselling, and education to standard survivorship care in a cohort of UGI and HPB cancer survivors who are > 3-months post-oesophagectomy/ gastrectomy/ pancreaticoduodenectomy, or major liver resection. One hundred twenty participants (60 per study arm) will be recruited to establish a mean increase in the primary outcome (cardiorespiratory fitness) of 3.5 ml/min/kg with 90% power, 5% significance allowing for 20% drop out. Study outcomes of physical function, body composition, nutritional status, HRQOL, and fatigue will be measured at baseline (T0), post-intervention (T1), and 3-months follow-up (T2). At 1-year follow-up (T3), HRQOL alone will be measured. The impact of ReStOre II on well-being will be examined qualitatively with focus groups/interviews (T1, T2). Bio-samples will be collected from T0-T2 to establish a national UGI and HPB cancer survivorship biobank. The cost effectiveness of ReStOre II will also be analysed.

**Discussion:**

This RCT will investigate the efficacy of a 12-week multidisciplinary rehabilitation programme for survivors of UGI and HPB cancer compared to standard survivorship care. If effective, ReStOre II will provide an exemplar model of rehabilitation for UGI and HPB cancer survivors.

**Trial registration:**

The study is registered with ClinicalTrials.gov, registration number: NCT03958019, date registered: 21/05/2019

## Background

With gradually improving survival rates, optimising the quality of upper gastrointestinal (UGI) and hepatopancreaticobiliary (HPB) cancer survivorship has come to the fore of UGI and HPB cancer research. Indeed, the need for rehabilitative strategies to counteract the multitudinous physical and nutritional side effects of UGI and HPB cancers and their treatments has increasingly been highlighted in the literature [1–3]. For potentially curative disease, surgical resection remains the mainstay treatment [4–6]. However, UGI and HPB surgery is inherently complex, and the associated risk of mortality and morbidity greatly exceeds that of other surgical procedures [7]. UGI and HPB resection leads to anatomical, and physiological changes in the GI tract resulting in significant issues with malnutrition and malabsorption post-operatively [8]. Furthermore, for locally advanced UGI and HPB cancers, a multimodality treatment approach, which combines surgery with neoadjuvant/adjuvant chemo/chemoradiotherapy, is favoured for its significant survival advantages compared to surgery alone [9, 10]. However, these treatments may precipitate further decrements in nutritional status [11]. Consequently, persistent weight loss and sarcopenia are ubiquitous in UGI and HPB cancer survivorship [3, 12], and in parallel there are prevailing impairments in physical function and health related quality of life (HRQOL) [1, 13–15]. Therefore, rehabilitative strategies that aim to minimize physical and nutritional deficits and in turn improve the health and well-being of survivors require exploration in this cohort.

Given the combined physical and nutritional challenges of UGI and HPB cancer survivorship, lifestyle interventions such as exercise and/or dietary rehabilitation are potential cost-effective strategies. Increasingly exercise interventions are advocated due to their positive effects on physical function, muscle strength, psychosocial status, and HRQOL [16, 17], and dietary programmes for their association with improvements in body weight and diet quality [18]. Therefore the potential benefits of such rehabilitative measures in UGI and HPB survivorship should not be underestimated. Moreover, increasingly UGI and HPB cancer survivors are reporting their need for, and willingness to engage in rehabilitation [19]. However, evidence supporting rehabilitation strategies in UGI and HPB cancer is currently lacking [2].

Preliminary work at this centre has established the safety, feasibility and initial efficacy of multidisciplinary rehabilitation in oesophago-gastric cancer survivorship [20–22]. The ReStOre (Rehabilitation Strategies following Oesophago-gastric Cancer) feasibility study and pilot RCT demonstrated that a 12-week programme of supervised and homebased exercise, 1:1 dietary counselling, and health education could result in clinically significant improvements in cardiorespiratory fitness [21, 22], and physical and mental well-being [23] without compromise to body composition. Thus the ReStOre RCT is the first evidence-based model of rehabilitation in UGI cancer survivorship. The ReStOre II RCT will now further examine the effectiveness of the ReStOre programme by RCT in a larger cohort of UGI and HPB cancer survivors.

## Methods

### Study aims

The primary aim of this work is to examine if a multidisciplinary cancer rehabilitation programme (ReStOre II), incorporating exercise and diet prescription, designed and tailored for disease-free survivors of UGI and HPB cancers, namely cancer of the oesophagus, stomach, pancreas and liver, can lead to improvements in cardiorespiratory fitness in comparison to standard survivorship care.

Secondary aims are;

- To examine the effect of the ReStOre II programme on physical functioning
- To determine the impact of the ReStOre II programme on body composition
- To explore the effect of the ReStOre II programme on dietary quality and nutritional status
- To examine the early and longer-term effects of the ReStOre II programme on patient reported outcomes including HRQOL, and fatigue
- To qualitatively examine the effects of the ReStOre II programme on physical, mental, and social well-being
- To evaluate the cost effectiveness of the ReStOre II programme
- To establish an UGI cancer survivorship biobank for collaborative translational research studies.

### Study design

**Fig. 1:**
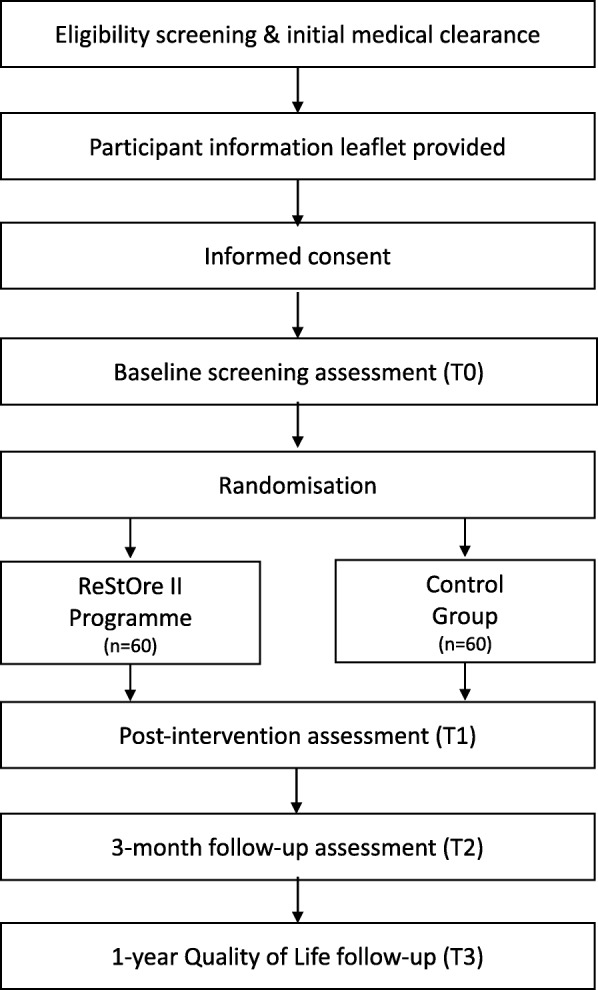
Participant flow through study

Using a convergent parallel mixed-methods study design, ReStOre II will be carried out as a randomised controlled trial with two arms: i) an intervention group offered the 12 week ReStOre II programme in addition to usual care, and ii) a control group receiving usual care. The flow of participants through the study is presented in Fig. 1. The study will recruit participants from three large teaching hospitals in Dublin, Ireland (St James’s Hospital, St Vincent’s University Hospital, and Tallaght University Hospital). Ethical approval has been granted from their respective research ethics committees and any subsequent amendments to the trial protocol will be submitted for their approval. The study will be conducted in accordance with the Declaration of Helsinki.

### Study participants

ReStOre II will recruit 120 patients with a histological confirmed diagnosis of cancer of the oesophagus, stomach, pancreas, or liver who have undergone surgery with curative intent. Participants must meet the following eligibility criteria; be ≥ three months post oesophagectomy, total gastrectomy, pancreaticoduodenectomy, or major liver resection, ± neo-adjuvant/adjuvant chemo/chemoradiotherapy with curative intent, and adjuvant therapy must be completed. Exclusion criteria are; ongoing serious post-operative morbidity, and, evidence of active or recurrent disease. In addition, those with any serious co-morbidity that would impact on exercise participation will be excluded, including those with; electrocardiograph (ECG) abnormalities at rest or during Cardiopulmonary Exercise Test (CPET), congestive heart failure (NY Heart Association Class II, III or IV), uncontrolled hypertension (resting systolic blood pressure > 180 mmHg and/or diastolic > 100 mmHg), recent serious cardiovascular events (within 12 months) including, but not limited to, cerebrovascular accident, and myocardial infarction, unstable cardiac, renal, lung, liver or other severe chronic disease, uncontrolled atrial fibrillation, or left ventricular function < 50%, %; and, severe/ very severe chronic obstructive disease (COPD) (GOLD Stages III/IV FEV1 < 50%, FEV1/FVC < 70%).

### Recruitment and screening

Members of the clinical team will determine potential participants from post-operative clinic lists and from hospital databases. Eligibility screening will be then be processed by the research team and research nurses. Written medical clearance from each participant’s treating consultant will be a pre-requisite to trial enrolment. Potential participants will receive a participant information leaflet (PIL) from a member of the study team. Following receipt of the trial PIL potential participants will then be given a one-week reflection period to consider their interest in trial participation. Upon completion of the reflection window, patients will receive a telephone call to establish whether they wish to participate. Those who express an interest in participation will be asked to attend a screening assessment in which they will provide written informed consent and complete baseline measures. This baseline assessment will take place in the Clinical Research Facility (CRF) at St James’s Hospital (SJH). If accrual is lower than anticipated, recruitment may also be expanded by advertising the study through charity partners; the Irish Cancer Society and the Oesophageal Cancer Fund.

### Randomisation and blinding

Following successful completion of baseline assessments, including a CPET, participants will be formally registered on the trial and will be randomised to the ReStOre II programme or the usual care control group. Block randomisation will be performed using a computer-generated randomisation list. Randomisation will be administered independently by the CRF at SJH. Study assessments will be performed by an assessor concealed to allocation. Given the design of the ReStOre II programme, it will not be feasible to blind either the research staff responsible for delivering the ReStOre intervention or trial participants to their allocation.

### Intervention

The ReStOre II intervention will take place in the exercise physiology suite at the CRF at SJH. The programme will follow a modified version of our established protocol from the ReStOre I study [22]. The ReStOre II programme comprises three elements: supervised and home-based exercise training, individualised dietetic counselling, and multidisciplinary education. The intervention is summarised in Table 1. In line with the Irish National Cancer Strategy 2017–2026 [24], key to the delivery of the programme will be an emphasis on self-management. At the start of the programme participants will set personal goals for the programme. Furthermore, each week participants will also set a specific personal goal for the coming week.

**Table 1:** The ReStOre II programme

Week	1	2	3	4	5	6	7	8	9	10	11	12
Supervised exercise sessions	×2	×2	×2	×1	×1	×1	× 1	× 1	–	× 1	–	× 1
Home based exercise	×1	×1	×2	×2	×3	× 3	× 3	× 3	×5	×4	×5	×4
1:1 Dietetic sessions	×1	×1		×1		×1		×1		×1		×1
Group education sessions	×1	×1	× 1	× 1		×1		×1		×1		×1

Depicts frequency of sessions per week

#### Exercise

The exercise component will consist of a 12-week supervised and home-based intervention. The exercise prescription will include both aerobic and resistance training and will be prescribed by a physiotherapist. Supervised group exercise sessions will be held twice weekly during the first 4 weeks to reintroduce exercise to participants in a safe and structured manner. As the programme progresses the frequency of supervised sessions will decrease, and the frequency of home-based exercise sessions will increase. This structure aims to encourage self-management in survivorship and increase autonomy with exercise prescription.

The ReStOre II exercise prescription is presented in Table 2. Aerobic training intensity will be individualised to the participant’s fitness. Exercise intensity will be prescribed using heart rate reserve (HRR) calculated using the Karvonen formula (HRR = maximum heart rate –resting heart rate) [25]. The values for maximum heart rate and resting heart rate will be calculated during the baseline CPET. Participants will wear Polar Heart Rate monitors to ensure compliance with the prescribed exercise intensity. Intensity will also be monitored with the Borg Perceived Scale of Exertion [26]. Upon completion of the ReStOre II programme, participants will be completing 150 min of moderate-vigorous intensity activity per week, as per ACSM physical activity guidelines [16]. Resistance training will also be tailored to the participant’s fitness levels. Specifically, the first week will be used to ensure safe and appropriate technique on all exercises and determine the training loads for subsequent sessions. The program will consist of 5 major movements (squat, lunge, hip flexion/extension, pushing and pulling) incorporating compound exercises targeting major muscle groups of the upper and lower body. Additionally, accessory movements targeting the biceps and triceps will be incorporated. Resistance will be added using free weights, resistance bands, a leg press machine, or body weight. Participants will be provided with resistance equipment for use at home. Where possible, loading will be progressed throughout the programme using the “2 for 2 rule” [27]. If an individual can complete two additional repetitions of an exercise, for 2 consecutive sessions, the weight for that exercise will be increased in the next session. We will target weight increases of ~ 5–10% for upper body exercises and 10–15% for lower body exercises [27]. In exercises where load can’t be added, participants will be asked to complete additional sets and/or reps of each exercise to ensure progression of training across the programme.

**Table 2:** ReStOre II exercise prescription

	Aerobic Training				Resistance Training			
	Frequency		Intensity	Time	Frequency		Intensity	Sets x Reps
	*Supervised Intervention*	*Home Exercise Programme*			*Supervised Intervention*	*Home Exercise Programme*		
Week 1	2	1	40–45% HRR	20	1	0	~16RM	1 × 12
Week 2	2	1	40–45% HRR	20	1	0	~14RM	2 × 12
Week 3	2	2	45–50% HRR	20	2	0	~14RM	3 × 12
Week 4	2	2	45–50% HRR	25	2	0	~14RM	3 × 12
Week 5	1	3	45–50% HRR	25	1	1	~12RM	3 × 10
Week 6	1	3	50–60% HRR	25	1	1	~12RM	3 × 10
Week 7	1	3	50–60% HRR	30	1	1	~11RM	4 × 10
Week 8	1	3	50–60% HRR	30	1	1	~10RM	3 × 8
Week 9	0	4	60–75% HRR	30	0	2	~10RM	3 × 8
Week 10	1	4	60–75% HRR	30	1	1	~8RM	3 × 6
Week 11	0	5	65–80% HRR	30	0	2	~7RM	4 × 6
Week 12	1	4	65–80% HRR	30	1	1	~12RM	2 × 8

*Abbreviations*: *HRR* heart rate reserve, *X1RM* X-repetition maximum, *Reps* number of repetitions

At each supervised session the exercise log will be reviewed by the physiotherapist, to monitor adherence and to facilitate exercise goal setting with the participant for the coming week. Adherence to the home-based exercise sessions will be monitored using Polar Heart Rate monitors and an exercise log. Adherence and compliance to resistance exercise component will be calculated using previously reported metrics [28]. Specifically, we will track what was initially prescribed, vs. what was actually achieved for each participant. These details and any deviations from the exercise protocol will be reported in the final manuscript. This programmme was chosen in accordance with our exercise facilities and resources available and has been utilised in prior studies with individuals with cancer [29, 30].

#### Dietary counselling

One-to-one dietetic sessions will be delivered during week 1, week 2 and fortnightly thereafter, or more frequently if required. Dietetic sessions will be delivered by a registered dietitian. Weight and circumferential measures will be recorded at each session and dietary intake will be assessed using tailored dietary interview strategies (incorporating 24-h recalls and qualitative information such as meal pattern and eating strategies). Nutritional requirements will be estimated using validated equations combined with appropriate stress and activity factors. The education delivered in the dietetic sessions will be individualised to participants’ needs, considering any dietary challenges such as dysphagia or malabsorption. The target for participants is to optimise dietary intake, ensuring adequate energy and micronutrient status, in alignment with the World Cancer Research Fund (WCRF) [31] and European Society for Clinical Nutrition and Metabolism (ESPEN) [32] guidelines for cancer survivors .

#### Multidisciplinary education

Education sessions (*n* = 7) will be delivered weekly during weeks 1–4 and fortnightly thereafter by a range of members of the multidisciplinary team including a doctor, dietitian, occupational therapist, and physiotherapist. Education topics will include an introduction to the ReStOre II programme and talks on items of pertinence to UGI and HPB cancer survivors including; benefits of physical activity, nutrition, management of ongoing medical issues in survivorship, fatigue management, and mindfulness.

### Standard care group

Participants in the control group will continue to receive standard care.

### Measures

ReStOre II study outcomes are listed in Table 3. The main assessment battery will be performed at; baseline (T0), post-intervention (T1), and 3-months post intervention (T2). Quality of life will be further assessed at 1-year post intervention (T3). At baseline information regarding socio-demographics will be collected from patient interview and data pertaining to medical history, cancer diagnosis and treatments will be obtained from patient’s medical records.

**Table 3:** ReStOre II study outcomes

Outcome	Instrument	Baseline	Post-intervention	3-month follow-up	1-year follow-up
		T0	T1	T2	T3
**Primary outcome**					
Cardiorespiratory fitness	Cardiopulmonary Exercise Test (CPET)	X	X	X	
**Secondary outcomes**					
Functional performance	Short Physical Performance Battery (SPPB)	X	X	X	
Muscle Strength	Leg Press 1-RM	X	X	X	
	Hand grip strength (HGS)	X	X	X	
Physical activity	Actigraph GT3X^+^ accelerometer	X	X	X	
Body composition	Anthropometry	X	X	X	
	Mid arm and waist circumference	X	X	X	
	Bioimpedance analysis	X	X	X	
Dietary intake	Dietary interview	X	X	X	
	Foodbook24	X	X	X	
Nutrition-related symptoms	Gastrointestinal Symptom Rating Scale (GSRS)	X	X	X	
	Simplified Nutritional Appetite Questionnaire (SNAQ)	X	X	X	
Quality of Life	EORTC-QLQ-C30	X	X	X	X
Cancer specific quality of Life	EORTC-QLQ-OG25 (oesophago-gastric cancer)	X	X	X	X
	EORTC-QLQ-HCC18 (liver cancer)	X	X	X	X
	EORTC-QLQ-PAN26 (pancreatic cancer)	X	X	X	X
Fatigue	Multidimensional Fatigue Inventory (MFI-20)	X	X	X	
Qualitative approach	Semi –structured interviews (focus groups or 1:1)		X	X	
Cost analyses	Clinical salaries, overheads and equipment costs				X
Blood samples	Serum, plasma and whole blood	X	X	X	
Adherence	Record in case report form/ exercise diary		X		
Other					
Sociodemographic details	Participant self-report	X			
Medical/ Cancer history	Medical records	X			
Adverse events	Reports of patients/ research personnel	X	X	X	X

#### Primary outcome - cardiopulmonary fitness

In ReStOre II, cardiopulmonary fitness, an important index of health [33], will be measured as the primary outcome during a maximal CPET. The CPET will be performed under medical supervision, using a ramp cycle ergometer protocol with breath-by-breath analysis (COSMED K4B^2^). The ramp gradient will be set to 10–25 watts/minute based on a calculation using predicted unloaded VO_2_, predicted VO_2_ at peak exercise, height, and age using the following standard equations [34].

1. VO_2_ unloaded in millilitres/minute (ml/min) = 150 + (6 x weight (kg))
2. Peak VO_2_ in ml/min = (height (cm) –age (years)) × 20(sedentary men) or × 14 (sedentary women)
3. Work rate increment minute/watts = ((peak VO_2_ ml/min – VO_2_ unloaded ml/min)/100)

Prior to test commencement, participants will undertake a 3 min warm-up of unloaded cycling. Breath-by-breath gas analysis, heart rate, ECG, blood pressure, oxygen saturation and blood lactate will be measured before, during and after testing. Testing will be terminated when the participant can no longer continue. Test termination will be followed by a 2 min cool down at a resistance of 30 watts, during which participants will be monitored for signs of distress. Peak oxygen uptake (VO_2_peak) will be calculated as the average value over the last 30 s of the test. Other values that will be recorded include; anaerobic threshold (lactate and ventilator threshold), peak work rate, peak heart rate and the respiratory exchange ratio.

#### Physical functioning

Physical functioning will be examined using a suite of validated objective measures examining functional performance, muscle strength, and physical activity. Functional performance will be determined using the Short Physical Performance Battery (SPPB). The SPPB is a reliable measure of physical functioning which consists of a gait speed, chair stand and balance test [35]. Scores range from 0 to 12, wherein a higher score indicates greater functional ability. Lower limb muscle strength will be measured by a 1-repetition maximum (1-RM) leg press test. The 1-RM is defined as the highest load that can be lifted through full range of movement at one time [36]. Participants will complete an appropriate aerobic and low intensity warm-up at 60% 1-RM and 80% 1-RM before a maximum of 5 trials to determine 1-RM. Hand grip strength (HGS) will be measured by handheld dynamometry. HGS provides a measure of hand and forearm strength and is found to correlate well with overall muscle strength and physical function [37]. For testing the participant will be seated, elbows at 90 degrees. Three attempts will be made on each hand with a 1-min rest between attempts. The highest value will be recorded. Physical activity levels will be measured by accelerometry using Actigraph GT3X^+^ activity monitors. The Actigraph GT3X^+^ is a well validated tool, used widely in oncology [38]. The small lightweight device will be worn at the hip for 7 days during waking hours to capture habitual physical activity. Data will be analysed with Actilife software using standardised algorithms to analyse time in physical activity domains (light, moderate and vigorous intensity) and adherence to ACSM physical activity guidelines (150 min moderate-to-vigorous intensity physical activity/week, accumulated in bouts ≥10 min) [36].

#### Body composition

Measures of body composition will include anthropometry and bioimpedance analysis (BIA). Weight (kilogrammes (kg)) and height (centimetres (cm)) will be recorded by standard methods as previously reported in the ReStOre feasibility study [21] and pilot RCT [22]. Body mass index (BMI) will be calculated as weight (kg)/ height (metres (m^2^)). Circumferential measurements (mid-arm muscle circumference and waist circumference) will be performed by standard procedures [39], taken in duplicate, and averaged for data entry. BIA will be used to determine body composition and will be performed using the SECA mBCA 515 (Seca, Hamburg, Germany). Measures recorded will include; fat mass, fat free mass, and skeletal muscle mass.

#### Dietary adequacy and nutrition related symptoms

Dietary intake and adequacy will be assessed by the study dietitian at T0, T1, and T2 using a structured dietary interview. In addition, for quantitative assessment, participants will complete a validated digital food frequency questionnaire, Foodbook24 [40]. Nutrition related symptoms will also be assessed using the validated Gastrointestinal Symptom Rating Scale (GSRS) [41], and the Simplified Nutritional Appetite Questionnaire (SNAQ) [42].

#### Quality of life

HRQOL will be determined by the European Organisation for Research and Treatment of Cancer Quality of Life Questionnaire (EORTC-QLQ-C30 version 3.0) and its relevant subscales. The EORTC-QLQ-C30 consists of functional scales (physical, role, cognitive, emotional, and social), symptom scales (fatigue, pain, nausuea and vomiting), global health status and HR-QOL scale, in addition to several single item symptom measures [43]. Cancer specific QOL issues will be assessed using the appropriate cancer subscale; QLQ-OG25 (oesophago-gastric cancer), QLQ-HCC18 (hepatocellular cancer), and QLQ-PAN26 (pancreatic cancer). To interpret the core questionnaire and cancer specific subscales, higher functional scores indicate greater functioning, whereas lower symptom scores indicate less symptom burden.

#### Fatigue

Fatigue will be measured using the Multidimensional Fatigue Inventory (MFI-20). The MFI-20 is a 20-item scale that measures the impact of fatigue in five dimensions: general, physical, cognitive, motivation and usual activities. It is scored from 0 to 20, with a cut-off score of ≥13 indicating severe fatigue. The psychometric properties of the MFI-20 have been tested and determined strong validity and reliability [44].

#### Qualitative data collection

Qualitative methods will be utilised to investigate intervention participants’ perceptions of the impact of the ReStOre II programme on their daily lives. Data will be collected through semi-structured focus group discussions immediately post-intervention (T1) and individual interviews at 3-months follow-up (T2). Focus groups at T1 will specifically explore the impact of the ReStOre II programme on mental, physical, and social well-being, whilst also examining the value of the group-based exercise programme and education talks to recovery. Individual interviews at T2 will focus more on examining the maintenance of health behaviours acquired during participation in the ReStOre programme. Interviews and focus groups will be led by a researcher experienced in qualitative methods and audio-recorded followed by verbatim transcription in preparation for data analysis.

#### Cost analysis

Programme implementation costs will be analysed in consideration of programme costs (e.g. clinician salaries, overheads and equipment costs). Changes in HR-QOL scores will be analysed and cost-effectiveness ratios calculated. The patterns of service use and related costs will be assessed for patients in each arm of the study, and alongside the costs of the intervention a comparison will be made of the total costs and outcomes, to estimated cost-effectiveness ratios.

#### Biosample collection

Serum, plasma, and whole blood samples will be collected at T0, T1, and T2 for the purpose of establishing a national UGI cancer survivorship biobank. Samples will be processed and stored at -80 °C at the Trinity Translational Medicine Institute, St James’s Hospital, Dublin 8 for future analyses to explore the mechanistic pathways underpinning the impact of multidisciplinary rehabilitation in survivorship.

#### Intervention Fidelity

In line with recent findings by Nilsen et al. [45], our research group also recognises the need to enhance reporting of adherence in exercise oncology trials. To this end, whilst also reporting on traditional adherence variables in our current research portfolio, we are now also endeavouring to report on additional variables adapted from drug trials in all our exercise oncology trials [45]. The ReStOre II trial will report on standard variables including supervised session attendance and the completion rate of home-based exercise sessions. Akin to our current PRE-HIIT trial examining high intensity interval training in advance of major thoracic surgery for cancer of the lung or oesophagus [46], ReStOre II will also include a number of novel adherence variables which are outlined fully in Table 4.

**Table 4:** Exercise adherence variables

Variable	Definition
Total number of supervised sessions attended	Total number of supervised sessions attended in the CRF at SJH
Total number of homebased sessions completed	Total number of homebased sessions reported in exercise diary as complete
Total number of compliant aerobic sessions completed	Total number of aerobic sessions (supervised/unsupervised) where prescribed aerobic exercise dosage was achieved
Total number of compliant resistance sessions	Total number of resistance sessions (supervised/unsupervised) where prescribed resistance training dosage was achieved
Permanent treatment discontinuation	Permanent discontinuation of the ReStOre II programme before week 12
Treatment interruption	Missing at least three consecutive supervised ReStOre II sessions
Dose modification	Number of supervised sessions requiring exercise dose modification
Early session termination	Number of supervised sessions requiring early session termination
Pre-treatment intensity modification	Number of supervised sessions requiring modification because of a pre-exercise screening indication.

### Safety

Patient safety will be paramount to the implementation of the ReStOre II trial. Standard safety measures will include; written medical clearance, and successful completion of a medically supervised CPET prior to trial commencement. All trial assessments and supervised exercise sessions will take place in the CRF which is located within the confines of SJH and is covered by their emergency response team. All adverse events will be documented, and serious adverse events will be communicated to the SJH/TUH and SVUH research ethics committees. Weight loss is a particular concern for UGI cancer survivors, and accordingly the study dietitian will monitor weight closely during the ReStOre II programme.

### Sample size calculation

Using data from the ReStOre pilot RCT [22] and supporting published literature [47], a sample size of 96 (48 per arm) is needed to detect a mean increase of 3.5 ml/min/kg in the intervention arm, assuming a 1.75 ml/kg/min increase in the control arm and standard deviation of change of 2.59 ml/kg/min for each arm, with 90% power at the 5% significance level based on a two-sample t-test. Given an expected attrition rate of 20%, a sample of 120 participants (60 per arm) will be recruited.

### Data management and analysis

The Data Management Plan will outline how research data will be handled during and after the project. Outcome assessments will be recorded in a paper-based case report form and then entered into a password protected computer data repository. Data validation will be used to avoid erroneous data entry. All participants will be allocated a unique study code. The key to the study code will be stored securely and separately. All paper records will be stored in locked filing cabinets, in a locked office in a restricted access building with swipe access. Electronic records will be stored on password protected encrypted devices. Upon completion of the trial an anonymised data set will be deposited on a secure online repository in line with open access publication requirements.

Quantitative data analysis will be completed using IBM SPSS software. Baseline characteristics will be presented for each study arm. Values for normally distributed continuous variables will be presented as means (standard deviations), whereas data which does not follow a normal distribution will be presented as median (range). Categorical variables will be displayed as counts and proportions. A linear mixed model will be used to model the longitudinal change in the primary response between the groups, allowing pair-wise deletion for missing data and allowing for within subject correlations in the repeated measures across time. The variance-covariance structure will be decided based on parsimony, and the ReStOre pilot RCT data. The model will inherently adjust for the baseline response variable. The primary endpoint will be the *p*-value for the interaction of time-point with treatment arm. The primary analysis set will comprise an Intention-to-treat group, including all patients with analysable data irrespective of fidelity, compliance, or arm cross-over. Statisticians will finalise a detailed Statistical Analysis Plan prior to the final T3 visit taking place and will remain blinded to study arm until the analysis is complete.

A qualitative descriptive approach [48] will be taken to the analysis of qualitative data. Braun and Clarke’s 6 stage approach to thematic analysis will be used to analyse all data collected [49]. A team of researchers will analyse all transcripts following an agreed process using nVivo 12 (QSR International, Australia).

### Trial management and governance

Management of the ReStOre II study will be overseen by three committees; a Trial management Group (TMG), Trial Steering Committee (TSC) and an Independent Data Monitoring Committee (IDMC). The TMG will oversee the daily trial management. The TSC will meet biannually and provide oversight of the trial and ensure the trial is conducted in accordance with the principles of Good Clinical Practice. The IDMC will monitor trial data to ensure the safety of the participants**.** The IDMC will meet biannually to review interim safety and accrual data. In addition, the IDMC may also meet at the discretion of the TSC.

### Dissemination

Findings of ReStOre II will be disseminated via peer-reviewed publications and conference presentations. Aggregate study results will be presented to participants and their families at an education symposium upon study completion. Anonymised data and all computer code used for the analyses will be made available on an open access repository.

### Public and patient involvement (PPI)

ReStOre II will involve a number of PPI initiatives. Firstly, a previous ReStOre participant is a collaborator on the trial and will sit on the TSC, and will review the study protocol, study procedures and documentation. Second, we have incorporated a PPI focused study within a trial (SWAT) into this study [50–53]. The SWAT will explore the effects of patient co-designed participant information on study recruitment rates. The protocol for the SWAT has been published separately [54]. Third, past ReStOre participants will attend the first class of each programme to meet and encourage new participants. Finally, a patient representative will be invited to speak at the education symposium.

### Study status

ReStOre II will begin in summer 2020.

## Discussion

The ReStOre II RCT, will investigate the efficacy of a 12-week multidisciplinary rehabilitation programme in UGI and HPB cancer survivorship. Whilst the initially feasibility of the programme is established in oesophagogastric cancer survivorship [21, 22], ReStOre II will now examine the programme as a definitive intervention in a wider cohort of UGI and HPB cancer survivors. The importance and novel nature of this body of work cannot be understated, particularly in view of the lack of evidence supporting rehabilitative strategies in UGI and HPB survivorship [2]. ReStOre II will be the first study to examine by RCT the impact of multidisciplinary rehabilitation following surgical resection for pancreatic or hepatocellular carcinoma. ReStOre II will include a 1-year follow-up evaluation of HRQOL, making it the first RCT to examine the longer-term impact of rehabilitation in UGI and HPB cancer survivorship. ReStOre II will also be the first to examine the cost-effectiveness of rehabilitation in UGI and HPB cancer survivorship. Furthermore, ReStOre II will establish a national UGI and HPB cancer survivorship biobank, allowing for innovative investigation of the underpinning biological effects of rehabilitation in the future.

A key strength of ReStOre II is the inclusion of cardiorespiratory fitness as the primary outcome. Cardiorespiratory fitness is a key predictor of health, cardiovascular and all-cause mortality, and HRQOL [55–57], and increasingly is highlighted as a strong predictor of cancer treatment outcomes [58], and survival [59, 60]. ReStOre II is further strengthened by its mixed methods approach. A key finding of the ReStOre pilot RCT was that there were many physical, mental, and social benefits to participation in the ReStOre programme that were not captured by HRQOL questionnaires, but captured instead by the post-intervention focus groups [22, 23]. Resultantly, ReStOre II will not only include post-intervention focus groups, but will also incorporate a 1:1 interview at 3-months post-intervention, to explore the longer-term effects of participation. This qualitative approach will provide substantial insight into the impact and acceptability of rehabilitation in UGI and HPB cancer survivorship. Moreover, the ReStOre II programme will also be enhanced by a greater focus on self-management strategies, including personalised goal setting. It is anticipated that this will aid participant’s sense of autonomy over their rehabilitation, and therefore optimise long-term adherence to lifestyle changes. ReStOre II will also benefit from the inclusion of PPI initiatives, especially the guidance of the patient collaborator, keeping the patient’s voice central to the ReStOre II study.

In conclusion, the ReStOre II RCT, will provide a model of rehabilitation for survivors of UGI and HPB cancer. This unique project addresses a clear gap in cancer research and will help inform much needed future clinical rehabilitative services for UGI and HPB cancer survivors.

## Data Availability

Not applicable.

## Abbreviations

**1-RM**: 1-Repition Maximum
**ACSM**: American College of Sports Medicine
**BIA**: Bioimpedance Analysis
**COPD**: Chronic Obstructive Pulmonary Disease
**CPET**: Cardiopulmonary Exercise Test
**CRF**: Clinical Research Facility
**ECG**: Electrocardiograph
**HGS**: Hand Grip Strength
**HPB**: Hepatopancreaticobiliary
**HRQOL**: Health Related Quality of Life
**HRR**: Heart Rate Reserve
**IDMC**: Independent Data Monitoring Committee
**PPI**: Patient and Public Involvement
**RCT**: Randomised Controlled Trial
**SJH**: St James’s Hospital
**SPPB**: Short Physical Performance Battery
**SWAT**: Study Within a Trial
**TSC**: Trial Steering Committee
**UGI**: Upper Gastrointestinal
